# Superior eyelid endoscopic transorbital approach to the tentorial area: A qualitative and quantitative anatomic study

**DOI:** 10.3389/fsurg.2022.1007447

**Published:** 2022-10-21

**Authors:** Andrea De Rosa, Alberto Di Somma, Alejandra Mosteiro, Abel Ferrés, Luis Alberto Reyes, Pedro Roldan, Ramon Torné, Jorge Torales, Domenico Solari, Luigi Maria Cavallo, Joaquim Enseñat, Alberto Prats-Galino

**Affiliations:** ^1^ Division of Neurosurgery, Department of Neurosciences, Reproductive and Odontostomatological Sciences, Università degli Studi di Napoli “Federico II”, Naples, Italy; ^2^ Department of Neurosurgery, Hospital Clinic, Barcelona, Spain; ^3^ Laboratory of Surgical Neuroanatomy, Faculty of Medicine, Universitat de Barcelona, Barcelona, Spain; ^4^ Research Group of Clinical Neurophysiology, Institut d’Investigacions Biomèdiques August Pi i Sunyer (IDIBAPS), Barcelona, Spain

**Keywords:** skull base, endoscopic, neurosurgery, brain tumors, basic and clinical research, transorbital approach

## Abstract

**Objective:**

Superior eyelid endoscopic transorbital approach (SETOA) is nowadays gaining progressive application in neurosurgical scenarios. Both anatomic and clinical reports have demonstrated the possibility of taking advantage of the orbital corridor as a minimally invasive route to reach anterior and middle cranial fossae and manage selected surgical lesions developing in these areas. The aim of this paper is to further shed light on other anatomic regions of the skull base as seen from a transorbital perspective, namely, the posterior cranial fossa and tentorial area, describing technical feasibility and steps in reaching this area through an extradural-transtentorial approach and providing quantitative evaluations of the “working area” obtained through this route.

**Material and methods:**

Four cadaveric heads (eight sides) were dissected at the Laboratory of Surgical Neuroanatomy (LSNA) of the University of Barcelona, Spain. A stepwise dissection of the transorbital approach to the tentorial area was described. Qualitative anatomical descriptions and quantitative analyses of working were evaluated by using pre- and postdissections CT and MRI scans, and three-dimensional reconstructions were made using Amira software.

**Results:**

With the endoscopic transorbital approach, posterior cranial fossa dura was reached by an extradural middle cranial fossa approach and drilling of the petrous apex. After clipping the superior petrosal sinus, the tentorium was divided and cut. An endoscope was then introduced in the posterior cranial fossa at the level of the tentorial incisura. Qualitative analysis provided a description of the tentorial and petrosal surfaces of the cerebellum, middle tentorial incisura, cerebellopontine fissures, and, after arachnoid dissection, by a 30° endoscopic visualization, the posterior aspect of the cerebellomesencephalic fissure. Quantitative analysis of the “working area” obtained after bone removal was also provided.

**Conclusions:**

This anatomic qualitative and quantitative study sheds light on the anatomy of the posterior cranial fossa contents, such as the tentorial area and incisura, as seen through a transorbital perspective. The first aim of the article is to enrich the anatomical knowledge as seen through this relatively new corridor and to provide quantitative details and insights into the technical feasibility of reaching these regions in a surgical scenario.

## Introduction

The endoscopic transorbital approach is nowadays entering the neurosurgical armamentarium as a minimally invasive approach for the management of selected skull base lesions, primarily involving the orbit and the anterior and middle cranial fossae (1–3). Its application ended up in clinical settings starting from several anatomic studies investigating the feasibility of such an approach and shedding light on a different perception of the anatomy of the skull base as seen through a ventrolateral perspective. The anatomy of the posterior cranial fossa, and particularly tentorial incisura, has been initially described through a transorbital perspective by utilizing an intradural transtentorial route (4), but a quantitative evaluation of the working space obtained with this corridor has not been reported. The aim of this article is to describe further the anatomy of the posterior cranial fossa and tentorial incisura, reached by an extradural transorbital-transtentorial corridor, and to add detailed quantitative measures of the working area, expressed as the total available space that is exposed after dedicated bone removal and incision of the tentorium, achieved by this minimally invasive route.

## Materials and methods

Anatomic dissections were performed at the Laboratory of Surgical NeuroAnatomy (University of Barcelona, Spain). Four specimens (eight orbits) were cleaned from blood clots, fixed with Cambridge solution, and injected with red and blue latex to highlight arterial and venous systems, respectively. Before and after dissection, all specimens underwent a multislice helical computed tomography (CT) scan (SIEMENS Somaton GoTop software version VA30A-SP03) with 0.5 mm thick axial spiral sections and a 0° gantry angle and an MRI study to obtain a 3D reconstruction of the main neurovascular structures. Endoscopic transorbital approaches were performed using a rigid endoscope of 4 mm diameter and 18 cm length, with 0° and 30° lenses (Karl Storz, Tuttlingen, Germany). The endoscope was connected to a light source through a fiber optic cable (300 W Xenon; Karl Storz) and to an HD camera (Endovision Telecam SL; Karl Storz). Data were uploaded to the Medtronic Workstation System to allow navigation guidance and point registration during dissection. A superior eyelid endoscopic transorbital approach was then performed as previously described (5–9).

### Endoscopic transorbital approach

**Figure 1:**
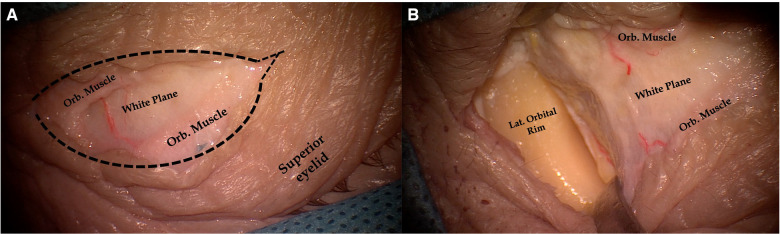
Pictures showing a skin incision for the endoscopic transorbital approach. The skin incision is run along a wrinkle of the superior eyelid. The orbicularis muscle is identified, and its fibers are separated, thus exposing the “white plane”, which is formed by the orbital septum and superior tarsus and which represents the posterior limit of the dissection (**A**). Dissection proceeds laterally until the periosteum of the lateral orbital rim is reached and cut. After the lateral orbital rim is completely skeletonized, subperiosteal–subperiorbital dissection can be started (**B**). Black dotted line, skin incision; Orb. Muscle, orbitalis muscle; Lat. Orbital Rim, lateral orbital rim.

The specimen was placed in line with the position of the head during the surgical procedure, i.e., with the head in a neutral position, with a slight flexion 5°–10° toward the contralateral side of the approach, to position the lateral wall of the orbit in an optimal position for drilling during the “working space creation” phase. The dissection was accomplished with a four-hand technique in which one surgeon usually holds the endoscope and the aspirator, and the other one performs the dissection manually. This is also the technique we usually apply during surgery, without the need for pneumatic or automatic endoscope holders. The skin phase of the procedure was accomplished with the aim of an operating microscope. A curvilinear incision along an eyelid wrinkle, extended about 1.5 cm laterally, was performed. The skin, subcutaneous tissue, and orbicularis muscle were divided until the “white plane,” made of the orbital septum and superior tarsus, was reached and respected (5). Dissection proceeded laterally to expose the lateral orbital rim, which was fully skeletonized. Temporalis muscle and fascia were detached from the lateral aspect of the orbital rim (Figure 1). Medially, the dissection was pursued in a subperiosteal fashion to detach the periorbita from the lateral wall of the orbit without violating it so that orbital fat tissue would not protrude into the surgical field. Zygomaticofacial and zygomaticotemporal arteries were identified and cut at the inferolateral border of the lateral wall of the orbit. Periorbita dissection proceeded until the inferior and superior orbital fissures were reached; at this point, the operative field was wide enough to allow the introduction of the endoscope. Drilling of the lateral wall of the orbit started until temporalis fascia was identified. Temporalis fascia and inferior and superior orbital fissures are the main anatomic landmarks in this phase, and their identification provides orientation during the drilling of the posterior portion of the lateral wall of the orbit, formed here by the greater sphenoid wing, to reach the ventral aspect of the middle cranial fossa finally. Drilling of the greater sphenoid wing proceeded until a “V”-shaped osseous wall is identified, limited medially by the sphenoid crest (10) (in the depth of the surgical field) and periorbita, laterally by the external surfaces of the greater sphenoid wing and temporal bone (in relationship with the temporalis muscle and fascia), inferiorly by the junction between the lateral wall and the floor of the orbit pointing at the inferior orbital fissure, and superiorly by the bone corresponding to the lesser sphenoid wing. Superomedially, the superior orbital fissure can be encountered.

### Extradural middle cranial fossa-transtentorial approach

**Figure 2:**
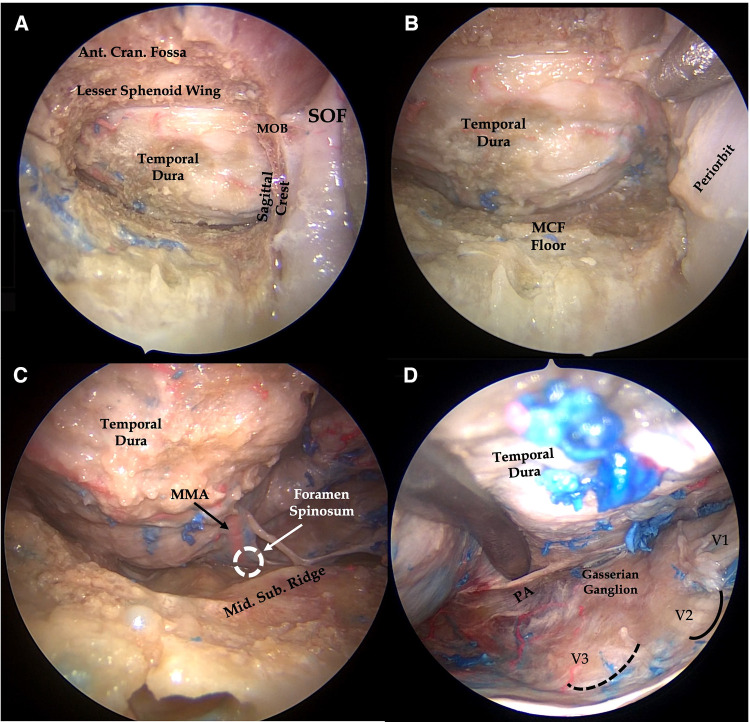
Right endoscopic transorbital approach to the middle cranial fossa. After removal of the anterior aspect of the greater sphenoid wing, the temporal dura is exposed (**A**) and peeled off from the middle fossa floor (**B**), which is further flattened. Once the foramen rotundum is exposed, peeling of the dura mater is started from V2, thus exposing the lateral wall of the cavernous sinus along with cranial nerves running within it. The middle meningeal artery, running through the foramen spinosum, is also identified and cut (**C**). Dura covering the middle fossa floor is then elevated posteriorly to expose the trigeminal ganglion and petrous apex (**D**). III, third cranial nerve; V1, ophthalmic division of trigeminal nerve; V2, maxillary division of the trigeminal nerve; V3, Mandibular division of trigeminal nerve; MCF Floor, middle cranial fossa floor; Mid. Sub. Ridge, midsubtemporal ridge; MMA, middle meningeal artery; MOB, meningo-orbital band; PA, petrous apex; black dotted line, foramen ovale; continuous black line, foramen rotundum.

Once the dura mater covering the temporal pole was exposed at the center of the “V”-shaped osseous wall, bone removal proceeded medially at the level of the sagittal crest, leading to the meningo-orbital band (11), which was cut. At this point, temporal dura was peeled off the middle cranial fossa lateral wall and floor, exposing the foramen rotundum with maxillary division of the trigeminal nerve (V2), anteriorly and inferomedially, and *eminentia arcuata*, posteriorly and laterally (12). Before that, the mid-subtemporal ridge came into view. Peeling of the lateral wall of the cavernous sinus started from V2, exposing the ophthalmic division (V1) of the trigeminal nerve and the oculomotor nerve, all entering the superior orbital fissure. Peeling continued until the trigeminal ganglion (inside the Meckel cave) and petrous apex were reached (Figure 2).

Drilling and flattening of the floor of the middle fossa were then completed by removal of the mid-subtemporal ridge, behind which foramen spinosum and foramen *ovale* were identified. The middle meningeal artery was cut. The anterolateral surface of the petrous bone, from *eminentia arcuata* to the petrous apex, was thus exposed. After identification of the main anatomical landmarks defining the Kawase triangle, namely, V3 and trigeminal ganglion, anteriorly, the greater petrosal nerve, laterally, and the *eminentia arcuata*, posterolaterally, and after gentle medialization of trigeminal ganglion and V3, drilling of the petrous apex was started as previously described to enter posterior cranial fossa (13). Drilling of the petrous apex represents an important step during this approach to improve surgical visualization and maneuverability within the posterior cranial fossa (13). This step also facilitates superior sagittal sinus detachment and clipping and the subsequent tentorial incision. At this point, the endoscope was entered through the drilled portion of the petrous apex toward the posterior cranial fossa to identify the vestibulofacial bundle entering the internal acoustic meatus. This allows for additional bone removal at the level of the roof of the internal acoustic meatus. Dissection proceeded with isolation and cut of the superior petrosal sinus along the superior petrous ridge, in its more medial aspect.

### Calculation of the working area

After the approach was completed and under endoscopic visualization, we used neuronavigation to collect coordinates delimitating the contours of the available space for surgical maneuverability within the middle and posterior cranial fossae, namely, the “working area.” Such coordinates were then transferred to Amira software, where they were fused with the pre- and postdissection CT scan of the specimen. Through this software, we could then quantify and obtain a 3D reconstruction of the area comprised within the points collected. The procedure was applied to each side of each specimen.

## Results

### Qualitative analysis

**Figure 3:**
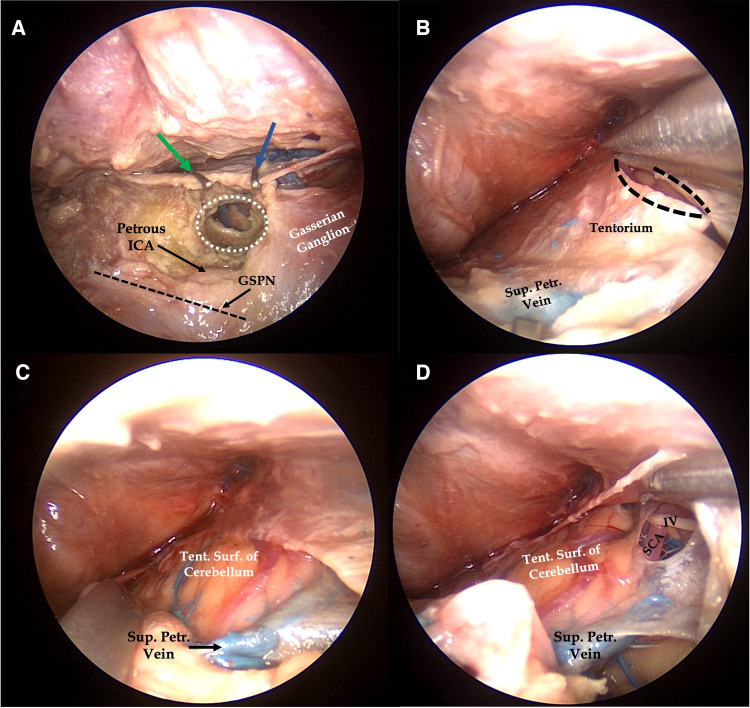
Right endoscopic transorbital approach to the tentorial incisura. After exposure of the petrous apex, two surgical clips (green and blue arrows) are positioned to the superior petrosal sinus before the tentorium cut. After identification of the anatomical landmark of Kawase's quadrilateral, the petrous apex is drilled (**A**). Tentorium cut is extended posteriorly from the area of the superior petrosal sinus closed by the surgical clips (**B**), thus exposing the tentorial surface of the cerebellum, laterally (**C**), and the middle tentorial incisura, medially (**D**). White dotted area, petrous apex removed after drilling; straight dark dotted line, course of the greater superficial petrosal nerve; curved dark dotted lines, margins of tentorium after the cut; IV, trochlear nerve; GSPN, greater superficial petrosal nerve; Petrous ICA, petrous segment of the internal carotid artery; SCA, superior cerebellar artery; Sup. Petr. Vein, superior petrosal vein; Tent. Surf. of Cerebellum, tentorial surface of the cerebellum.

**Figure 4:**
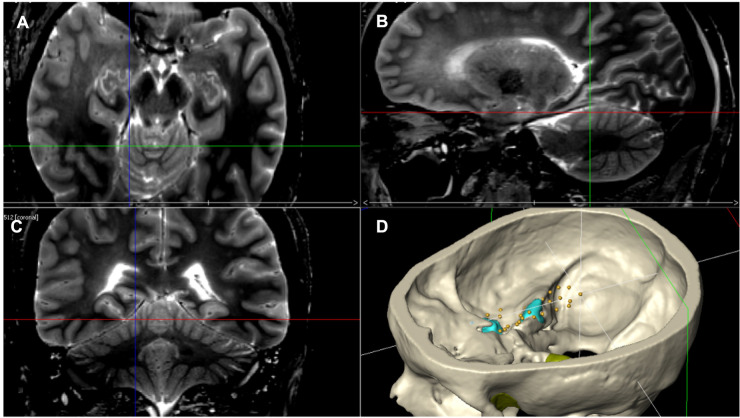
Pictures retrieved from neuronavigation axial (**A**), sagittal (**B**), and coronal (**C**) T2 MRI scans of one specimen and from Amira software reconstruction (**D**) of the same specimen indicating the posterior limit of the tentorial cut.

Once the superior petrosal sinus was identified and dissected from the superior petrosal ridge, two surgical clips were positioned, from lateral to medial, starting from right medial to the level of the internal acoustic meatus. Tentorium was cut starting in between the two surgical clips, given that the trochlear nerve was expected to enter the free margin of the tentorium far more anteriorly. The tentorial incision was then extended in a posterior and medial direction (Figures 3, 4).

**Figure 5:**
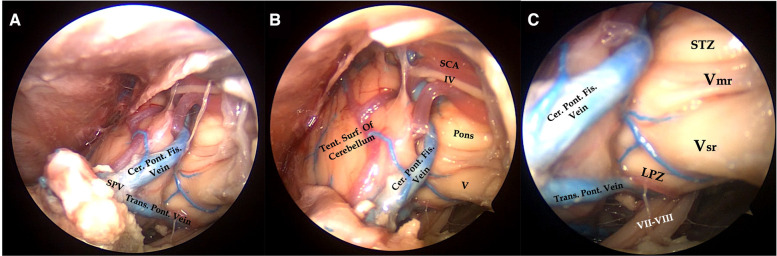
Right endoscopic transorbital approach to the tentorial incisura. After superomedial retraction of the margin of the tentorium, the contents of the middle incisural space are exposed (**A**). Cisternal segment of the trochlear nerve running along the superior surface of the superior cerebellar artery, lateral surface of the pons, the origin of the trigeminal root, and vein of the cerebellopontine fissure joining to the superior petrosal vein are visualized (**B**). By means of a 30° lens, directing the endoscope inferomedially, the vestibulofacial bundle is exposed in its course to the internal acoustic meatus, along with the transverse pontine vein joining the superior petrosal vein (**C**). IV, trochlear nerve; V, trigeminal nerve; Vsr, sensory root of the trigeminal nerve; Vmr, motor rootlets of the trigeminal nerve; VII–VIII, vestibulofacial bundle; Cer. Pon. Fis. Vein, Cerebellopontine fissure vein; LPZ, lateral pontine zone; SCA, superior cerebellar artery; SPV, superior petrosal vein; STZ, supratrigeminal zone; Tent. Surf. of Cerebellum, tentorial surface of the cerebellum; Trans. Pon. Vein, transverse pontine vein.

After the tentorium incision, the tentorial surface of the cerebellum, along with terminal branches of the superior cerebellar artery, came into view. At the most anterior part of the field, the superior petrosal vein, or Dandy vein, and its junction with the superior petrosal sinus could be visualized. By detaching and elevating the tentorium medially, the tentorial incisura, and particularly the middle incisural space below the free tentorial edge, corresponding to the ambient cistern, could be exposed. After arachnoid dissection, the cisternal portion of the trochlear nerve, running along the superior margin of the main trunk of the superior cerebellar artery, came into view. Below the superior cerebellar artery, the ponto-mesencephalic fissure was visualized and, at the most anterior and medial part of the field, the trigeminal root, emerging from the mid part of the pons, appeared with its superior and lateral direction, before leaning on the trigeminal impression of the petrous bone and entering the Meckel cave in the middle fossa. The transverse pontine vein, in relation to the emerging root of the trigeminal nerve, and the vein of cerebellopontine fissure were also appreciated. With a 30° endoscope, by directing the light inferomedially, the vestibulofacial bundle was highlighted (Figure 5).

**Figure 6:**
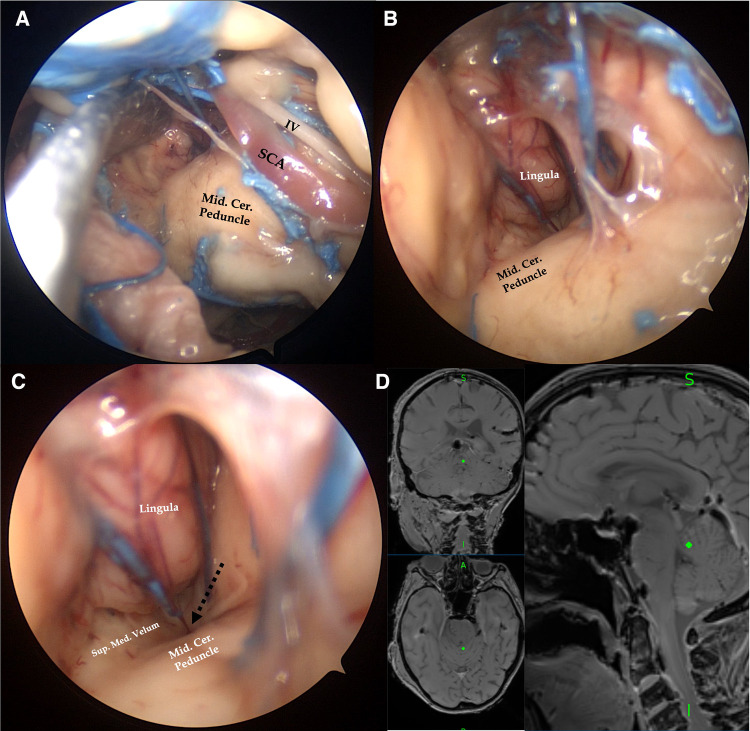
Right endoscopic transorbital exposure of the tentorial incisura and cerebellomesencephalic fissure. Extending forward arachnoid dissection of the cerebellopontine fissure, the middle cerebellar peduncle is divided from the petrosal surface of the cerebellum (**A**). By inserting a 30°endoscope along the superior margin of the middle cerebellar peduncle and directing the light inferiorly, the posterior margin of the cerebellomesencephalic fissure, represented by the lingula of the cerebellum, and the external surface of the superior part of the roof of the fourth ventricle (black dotted arrow), represented by the superior medullary velum, are visualized (**B,C**). We confirmed our anatomic findings by pointing the navigator (Medtronic StealthStation) at the level of the lingula (**D**). IV, cisternal portion of the trochlear nerve; Mid.Cer.Peduncle, middle cerebellar peduncle; SCA, superior cerebellar artery; Sup.Med.Velum, superior medullary velum.

We pushed our arachnoid dissection forward by opening the cerebellopontine cistern, limited laterally by the petrosal surface of the cerebellum and medially by the middle cerebellar peduncle. By dividing the anteromedial border of the cerebellum laterally and moving a 30° endoscope in the deep of the cistern, along the upper border of the middle cerebellar peduncle, the posterior-most aspect of the cerebellomesencephalic fissure was reached. In this space, we could evaluate the posterior margin of the fissure, limited by the lingula of the cerebellum, which lies above the superior medullary velum. This region corresponds to the cisternal (external) surface of the upper part of the roof of the fourth ventricle (Figure 6) (Video 1).

### Quantitative analysis

**Figure 7:**
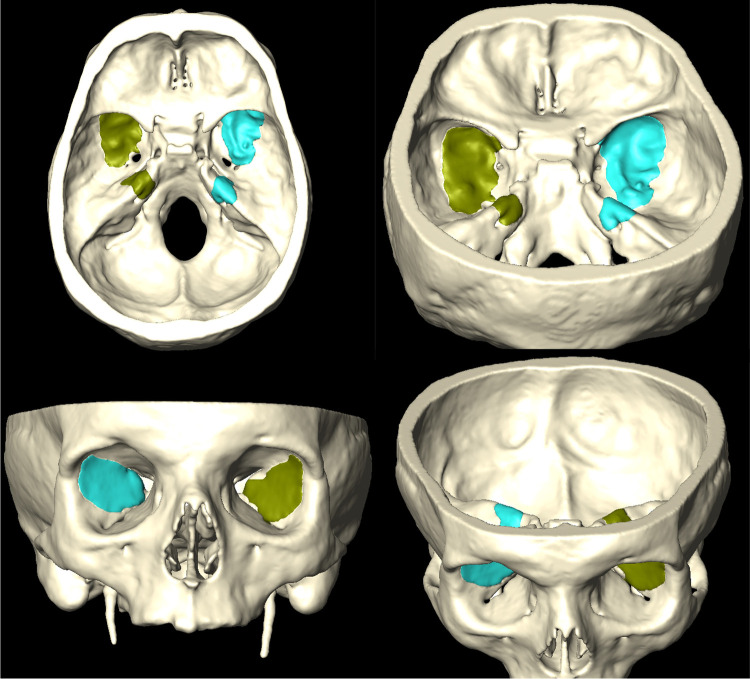
Amira software 3D reconstructions from postdissection CT scans, highlighting the amount of bone removal at the level of the middle cranial fossa and petrous apex.

**Figure 8:**
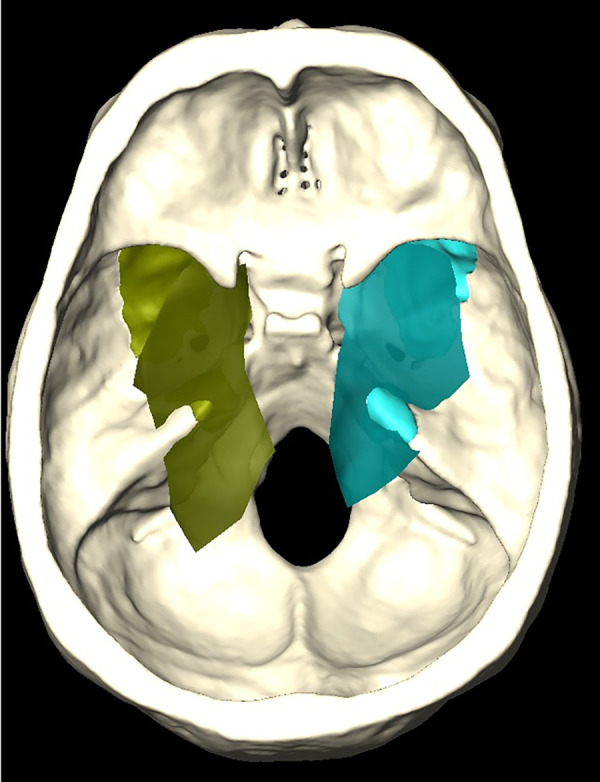
Amira software 3D reconstruction showing the working area (superimposed shaded green and blue areas) at the level of the middle and posterior cranial fossae, which can be obtained after bone removal (darker underimposed green and blue areas).

The working area was defined as the available surface of surgical maneuverability after extensive bone drilling of the middle cranial fossa floor and of the petrous apex, and tentorial incisions were achieved. This area includes a larger amount of the middle cranial fossa and a lesser amount of the posterior cranial fossa, which is reached out after tentorial incision and division. We obtained eight measures from our four specimens (Table 1), and the mean value was 2,800.52 mm^3^ (SD ± 364.28) (Figures 7, 8).

**Table 1:** Quantitative analysis of the available “working area” within the posterior and middle cranial fossa, obtained after complete bone removal through the endoscopic transorbital approach.

Specimen	Side	Working area (mm^3^)
1	Right	3,432.64
	Left	2,296.15
2	Right	2,916.45
	Left	2,726.27
3	Right	3,009.62
	Left	2,422.02
4	Right	2,986.30
	Left	2,614.74
Mean (mm^3^)		2,800.52
Standard deviation		±364.28

## Discussion

Since its introduction, in the last 10 years, transorbital endoscopic surgery has been increasingly utilized in the neurosurgical field as an alternative to standard craniotomies to manage selected lesions of the skull base (8). In its early applications, this minimally invasive corridor was proposed as an adjunct to the well-known endoscopic endonasal corridor to reach the lateral-most portion of central skull base lesions with large parasellar extension (14). Anatomic studies have helped in improving the knowledge of anatomy as seen through the ventral perspective provided by the transorbital route: anterior and middle cranial fossa compartments, along with the optocarotid region, sylvian fissure, cavernous sinus, Meckel cave, and petrous apex, have been extensively described (10, 11, 13, 15–20). As neurosurgeons became more familiar with this new anatomic perspective, surgical applications and indications of the endoscopic transorbital approach, alone or in combination with other minimally invasive corridors, namely, the endonasal one, extended to the removal of several skull base lesions, both intradural and extradural, such as intraorbital lesions, intraparenchymal temporal lesions, cavernous sinus meningiomas, trigeminal schwannomas, spheno-orbital meningiomas, middle cranial fossa meningiomas, and petroclival meningiomas (6, 7, 21–27). Recently, as a resume to the collected pearls and pitfalls of this approach, a classification of the levels of difficulty of the endoscopic transorbital approach for the management of different lesions has been proposed, pointing out the steps of the learning curve that the neurosurgeon must gain to minimize complications and achieve better patient outcomes (28). Tentorial incisura is one of the most challenging regions to reach, and, given the variety of its content, it could be the site of many kinds of lesions, such as trigeminal schwannomas, petroclival and tentorial meningiomas, and brainstem lesions. Many transcranial approaches are nowadays considered the gold standard to gain access to this region; among the others are the anterior and posterior petrosal, occipital-transtentorial, and supracerebellar-infratentorial approaches together with their modifications (29–36).

In a recent paper by Vasquez et al. (37), four different transcranial approaches, namely, frontotemporal transsylvian transtentorial (38, 39), subtemporal transtentorial (40, 41), posterior petrosectomy (42), and combined posterior suprainfratentorial transsinus approaches (43), addressed to the tentorial region and with the common step of splitting the tentorium to gain a wider visualization, were compared in terms of area of exposure. The transsylvian transtentorial approach provided a limited area of visualization of the most anterior portion of the tentorial incisura, with the potential complication of injury to the oculomotor nerve along its course under the tentorium. The subtemporal approach, with the addition of petrosectomy as described by Kawase, provides a wider exposure of the middle tentorial incisura, after tentorium is cut, with the main disadvantages represented by the retraction of the temporal lobe. With the posterior petrosectomy, the area of visualization is further enhanced because exposure from the midbrain, superiorly, to the lower cranial nerve, inferiorly, is possible but with the risk of injury to the venous sinuses and vein of Labbé, to the labyrinth and cochlea, and still to the temporal lobe because of sustained retraction. The combined posterior supra/infratentorial-transsinus approach allows for a wide visualization of the posterior portion of the tentorial incisura and pineal region, with the main drawback being represented by the need for transecting the nondominant transverse sinus during the approach (37).

In this scenario, we aim to describe the anatomy of the tentorial incisura through an endoscopic extradural-middle cranial fossa-transtentorial transorbital approach and quantify the amount of available working area that is obtained after extensive bone work and tentorial incision. Middle tentorial incisura could be reached in a completely extradural fashion, and extensive drilling of the middle fossa floor and the petrous apex provided the surgical maneuverability to detach and clip the superior petrosal sinus and then open the tentorium. Tentorial incision and opening can be achieved away from the entry point of the trochlear nerve. With a 0°-lens endoscope, the inferior portion of the middle tentorial incisura, corresponding to the ambiens cistern, can be visualized. The surgical exposure is limited medially by the trigeminal root, superiorly by the free edge of the tentorium, which also limits the exposure of the superior compartment of the middle incisura, laterally by the tentorial surface of the cerebellum, and inferiorly by a plane parallel to the petrous bone. We observed that surgical maneuverability could be furtherly improved by drilling the superolateral aspect of the petrous apex, corresponding to the roof of the internal acoustic meatus, a procedure that must be accomplished only when vestibulocochlear and facial nerves are identified in their course to the internal acoustic meatus (suprameatal drilling). The lateral surface of the mesencephalon and pons, along with the origin of the trigeminal nerve, the superior cerebellar artery along with the trochlear nerve crossing the ambiens cistern, can be exposed. With the aim of a 30°-lens endoscope, pointing inferomedially, the prepontine cistern can also be visualized. Furthermore, arachnoid dissection between the petrosal surface of the cerebellum and pons allows for the opening of the cerebellopontine fissure, which can be entered following the superior aspect of the middle cerebellar peduncle until the dorsal aspect of the pons is reached. This region corresponds to the cerebellomesencephalic fissure, limited posteriorly by the lingula of the cerebellum, inferiorly by the superior medullary velum, and anteriorly by the posterior surface of the midbrain.

Recently, Lin et al. already described the anatomy of the middle tentorial incisura through an intradural transsylvian transorbital approach (4). In their article, the middle tentorial incisura is reached after exposure of both anterior and middle cranial fossa and resection of the anterior clinoid. Then, after the temporal dural incision, an intradural corridor between the cavernous sinus, medially, and the medial temporal lobe, laterally, is used to gain access to the tentorial incisura. We propose a different pathway to reach this region in a complete extradural fashion, thus limiting the manipulation of the temporal lobe intradurally and avoiding the dissection of the arterial vasculature around the mesial temporal lobe. However, a more anterior exposure of the crural and interpeduncular cistern, which was described by the authors, was not exposed with our corridor.

Even though our contribution is a purely anatomic description, we think that, according to the results of quantitative analysis of the working area obtained after the approach, some considerations can be made regarding the clinical applicability of the transorbital route directed at the tentorial incision. Because of the working space that can be obtained if extensive drilling of the middle fossa floor and petrous apex is achieved, tumors extending from the middle fossa and projecting into the middle tentorial incisura, such as meningiomas arising from the dura mater surrounding the region of the tentorial incisura, with a medial and inferior extension could be reached with this kind of approach (probably in combination with the endonasal route). Furthermore, if limited, brainstem exposure also provides access to two safe entry zones of the pons (44), namely, the lateral pontine zone and supratrigeminal zone. Compared to the more conventional transcranial route, the advantages of this approach would be represented by minimal soft tissue manipulation, the absence of a visible scar (performing the skin incision in a wrinkle of the superior eyelid allows to hide the surgical wound when the patient is with opened eyes), which translates in a better aesthetic outcome for the patient, and the minimal temporal lobe retraction needed to reach the tentorial area. On the other hand, lesser surgical maneuverability provides a remarkable drawback in managing venous or arterial bleeding, a concept shared with any endoscopic skull base technique. Nevertheless, we are aware that clinical experience with the transtentorial extension of the endoscopic transorbital approach is still lacking and it would require great surgeon's expertise and specific instrumentation to be safely perfromed; endoscopic transorbital surgery in the posterior cranial fossa is indeed classified as the final step (Level 5 of difficulty) in the classification recently proposed (28). It must also be stressed that even if this corridor provides a minimally invasive alternative to standard craniotomies, thus avoiding complications related to these approaches such as brain retraction and contusion, venous infarction, soft-tissue complications related to craniotomy, the improving clinical experience suggests that ophthalmologic and visual complications remain one the main concerns of this surgical strategy, even if permanent visual alterations have been rarely described until now (45). Concerning cerebrospinal fluid leak, which is one of the main complications related to the endoscopic endonasal corridor, does not seem to be a frequent sequela of the transorbital route since, at the end of the procedure, tight closure can be achieved for all the tissue layers.

### Study limitations

We are aware that, as it happens with cadaveric anatomical study, differences in tissue consistency, the absence of bleeding, which could be present, for example, during middle cranial fossa floor drilling or during tentorium cut and elevation, and the tolerance to retraction all make anatomic results difficult to translate into clinical applications. Our contribution should be interpreted as an adjunct to the available knowledge of anatomy as seen from this ventral perspective. Furthermore, the design of our article was not intended to provide a comparison between the perspectives given by the endoscopic transorbital approach and other transcranial approaches that are used to reach the tentorial area. We think that such a comparison would have added important information about the pros and cons of different surgical strategies. Future studies are already planned to address this issue.

## Conclusions

In this article, we provided an anatomical description of the middle incisural space as seen through an endoscopic transorbital perspective and a quantification of the working area that can be obtained. In particular, we propose a purely extradural-middle cranial fossa approach to reach and cut the tentorium. We observed that adequate “bone work” on the middle cranial fossa floor and petrous apex provides adequate working space for surgical maneuverability in this deep-seated region of the skull base. Further anatomical contributions, comparing this corridor to the standard transcranial approaches, and clinical reports are necessary to highlight the possible advantages and indications of this technique.

## Data Availability

The raw data supporting the conclusions of this article will be made available by the authors, without undue reservation.
